# Description of the Quality of Life of Patients With Subarachnoid Hemorrhage at King Abdulaziz University Hospital in Jeddah

**DOI:** 10.7759/cureus.43164

**Published:** 2023-08-08

**Authors:** Ahad Tariq Banjar, Mohammed Alyousef, Nidhal Mohammad Almohammady, Reem Nezar Almustafa, Raghad Khalid Alotaibi, Medaa Yasir Hijji, Lujain Mohammed Filimban, Mariyah Ghassan Mousa

**Affiliations:** 1 Department of Medicine, King Abdulaziz University, Jeddah, SAU; 2 Department of Neurological Surgery, King Abdulaziz University Faculty of Medicine, Jeddah, SAU; 3 Department of Medicine, King Abdulaziz University Faculty of Medicine, Jeddah, SAU

**Keywords:** mgos, quality of life, prognosis, modified glasgow outcome scale, subarachnoid hemorrhage

## Abstract

Objective

Stroke is a serious medical condition that causes long-term morbidity and disability. There are two types of stroke, i.e., ischemic and hemorrhagic stroke. Subarachnoid hemorrhage (SAH) accounts for 5% of all stroke cases worldwide. Stroke survivors may experience cognitive dysfunction in many forms. Evidence regarding the quality of life (QoL) of patients post-SAH in the Middle East is limited. Therefore, this study aims to describe the quality of life in patients with SAH at the King Abdulaziz University Hospital (KAUH) in Jeddah, Saudi Arabia, from April 2021 to October 2021.

Methods

We included patients who were diagnosed with SAH ≤ 10 years prior at our hospital and were admitted within 72 hours of the ictus. Patients were included using non-probability convenience sampling without randomization. We collected the Glasgow Coma Scale (GCS), World Federation of Neurosurgeons (WFNS), and Modified Glasgow Outcome Scale (MGOS) scores.

Results

We included 48 patients (mean age: 49.78 ± 19.44 years, male proportion: 62.5%). More than 50% of the participants had comorbidities. The mean baseline GCS, WFNS, and MGOS scores at admission were 12.62 ± 3.56, 2.19 ± 1.54, and 3.58 ± 1.67, respectively. Women had significantly higher MGOS scores than men (p ≤ 0.05). Death was significantly associated with low MGOS scores (p ≤ 0.05). Age showed a non-significant negative correlation with the MGOS score (r = - 0.17, p-value = 0.24). Finally, the MGOS score was significantly correlated with the baseline GCS and WFNS scores at admission (r = 0.68 and r = - 0.67, respectively).

Conclusion

Our findings demonstrated that a low MGOS score, which indicates more comorbidities, greatly affects the quality of life of patients with SAH. Moreover, the baseline GCS score was the best prognostic predictor for patients with SAH.

## Introduction

Stroke is a serious medical condition that causes long-term morbidity and disability. Worldwide, stroke is the second and third leading cause of mortality and disability, respectively [1]. Accordingly, it is important to assess and improve the quality of life (QoL) of stroke survivors. There are two types of stroke: ischemic and hemorrhagic. Subarachnoid hemorrhage (SAH) accounts for five percent of all stroke cases worldwide [2]. Stroke survivors may experience cognitive dysfunction, which often involves problems with memory, concentration, attention, or executive tasks; they may also experience emotional problems, including depression, anxiety, or post-traumatic stress disorder [3].

SAH is considered among the less common types of hemorrhagic strokes [4]. SAH is symptomatically characterized by the sudden onset of extremely severe headache, with accompanying neck stiffness, nausea, vomiting, photophobia, and brief loss of consciousness [5]. Blood accumulation in the subarachnoid space increases the cerebral pressure, thus interfering with brain function [6].

In 2010, approximately 7.9 per 100,000 persons per year suffered from SAH worldwide [7]. However, the health-related quality of life (HRQoL) of patients with SAH remains unclear. Aneurysmatic SAH (aSAH), which results from aneurysmal rupture, occurs at the age of 50-55 years [8] and is predominant among African Americans [9]. Moreover, the incidence rate of aSAH is slightly higher among women than among men due to hormone-replacement therapy [10]. Elucidating the predictors of HRQoL in patients with SAH could inform preventative and rehabilitation measures in health care [11]. A previous systematic review reported that being female is correlated with worse HRQoL, as is being single or divorced [12].

In Europe, several studies have demonstrated a decline in health after SAH. For example, a Spanish study reported a decrease in the physical condition of patients with SAH [13]. A German study reported a significant decrease in the HRQoL of patients with SAH compared with the general population, where the independent predictors of decreased HRQoL included female gender, severe SAH, functional disability, and depression [14].

A Turkish study reported a significantly decreased QoL (measured using the Montreal Cognitive Assessment and Short Form Health Survey) in patients with aSAH compared with the general population [15]; moreover, 60% and 25% of SAH survivors presented with cognitive dysfunction and low QoL, respectively. Although epilepsy could also decrease QoL, this was not investigated in the study. With respect to the gray-white-matter ratio, there were only slight gender differences in QoL. An Iranian study on cognitive function, depression, and QoL in patients with aSAH reported that 57% and 55% of the patients developed cognitive impairment and depression, respectively. Additionally, age was positively correlated with the risk of post-SAH cognitive impairment, and SAH had long-term effects including, but not limited to, the inability of patients to return to work and integrate into society [16].

Unfortunately, evidence regarding SAH in the Middle East remains unclear. Moreover, studies on HRQoL have mainly focused on patients with aSAH and long-neglected non-aneurysmal SAH. In this study, we aimed to describe the QoL in patients with SAH at King Abdulaziz University Hospital (KAUH) in Jeddah, Saudi Arabia, from April 2021 to October 2021.

## Materials and methods

We conducted a retrospective study at KAUH, Jeddah, Saudi Arabia, a tertiary healthcare center. We included patients with SAH, regardless of age, who visited the hospital between January 1, 2010, and December 31, 2020, from the KAUH Phoenix Database using ICD-10 codes related to SAH (I60.9 & S06.6). We included 48 eligible patients (18 women and 30 men) through non-probability convenience sampling without randomization according to the inclusion and exclusion criteria.

The inclusion criteria were as follows:

- Diagnosed with SAH within ≤10 years at KAUH - Hospital admission within 72 h of ictus - An elapsed period of ≥1 year from the time of diagnosis to allow for recovery.

The exclusion criteria were:

- Pregnancy during ictus - Previous neurodegenerative or psychiatric illnesses.

We collected data regarding patient demographics, smoking history, comorbidities, initial Glasgow Coma Scale (GCS) score (Table 1), World Federation of Neurosurgeons (WFNS) score at admission (Table 2), aneurysm location, death, and Modified Glasgow Outcome Scale (MGOS) scores (Table 3). The MGOS assesses neurological outcomes on a scale ranging from 0 to 5, where grades 0, 1, 2, 3, 4, and 5 indicate death with unknown cerebral status, death due to documented hypoxic brain damage, persistent vegetative state, severe disability, moderate disability, and mild/no disability, respectively [17].

**Table 1:** Glasgow coma scale

Behavior	Response	Score
Eye Opening Response	Spontaneously	4
	To speech	3
	To pain	2
	No Response	1
Best Verbal Response	Oriented to time, place, and person	5
	Confused	4
	Inappropriate words	3
	Incomprehensible sounds	2
	No response	1
Best Motor Response	Obeys commands	6
	Moves to localized pain	5
	Flexion withdrawal from pain	4
	Abnormal Flexion (decorticate)	3
	Abnormal Extension (decerebrate)	2
	No Response	1
Total Score	Best Response	15
	Comatose client	8 or less
	Totally unresponsive	3

**Table 2:** World Federation of Neurosurgeons

Grade	Glasgow Coma Scale	Motor Deficit
1	15	Absent
2	14-13	Absent
3	14-13	Present
4	12-7	Present or absent
5	6-3	Present or absent

**Table 3:** Modified Glasgow Coma Scale N.B.: MGOS = Modified Glasgow Outcome Scale; CT = Computerized Tomography; MRI = Magnetic Resonance Imaging; SSEP = Somatosensory Evoked Potential

MGOS	Neurological outcome
MGOS 0	Patient died with unknown cerebral status (e.g., death after cardiogenic/septic shock, pulmonary embolism, or aortic dissection without assessment of neurological status by cranial CT/MRI, SSEP, etc.)
MGOS 1	Patient died with documented hypoxic brain damage
MGOS 2	Persistent vegetative state (unable to interact with environment)
MGOS 3	Severe disability (unable to live independently but able to follow commands)
MGOS 4	Moderate disability (able to live independently but unable to return to work)
MGOS 5	Mild or no disability (able to return to work)

This study was approved by the Research Ethics Committee of KAUH (Reference no. 243-21), Jeddah, Saudi Arabia. We performed statistical analyses using Statistical Package for the Social Sciences (SPSS) version 26 (IBM Corp., Armonk, NY, USA). We present qualitative variables as numbers and percentages. We analyzed relationships between variables using the chi-squared test (χ2). We presented quantitative data as means ± standard deviations, which are analyzed using the Mann-Whitney and Kruskal-Wallis tests. We investigated correlations between variables through Spearman’s correlation analysis. We set statistical significance at p<0.05.

## Results

As shown in Table 4, the mean age of the included patients was 49.78 ± 19.44 years. Moreover, 62.5%, 72.9%, 54.2%, and 14.6% of the patients were male, non-Saudi nationalities, married, and smokers, respectively. As shown in Table 5, 58.3% of the participants had comorbidities, with hypertension (37.4%), diabetes mellitus (16.7%), and cardiovascular disease (14.6%) being the most common comorbidities. The mean initial GCS and WFNS scores at admission were 12.62 ± 3.56 and 2.19 ± 1.54, respectively. Some patients (43.8%) had aneurysms, with the most common aneurysm location being the anterior communicating artery (25%). Additionally, 22.9% of the patients underwent cerebral angiography with coiling, whereas 33.3% did not require surgical intervention. The mean MGOS score was 3.58 ± 1.67, and 20.8% of the patients died.

**Table 4:** Distribution of included patients according to their demographic data and smoking status. NA: not applicable; GCS: Glasgow Coma Scale; WFNS: World Federation of Neurosurgeons

Variable	No.	(%)
Age	49.78 ± 19.44	
Gender		
Female	18	37.5
Male	30	62.5
Nationality		
Saudi	12	25
Non-Saudi	35	72.9
NA	1	2.1
Marital status		
NA	11	22.9
Married	26	54.2
Single	11	22.9
Smoking history		
NA	9	18.8
No	32	66.7
Yes	7	14.6

**Table 5:** Distribution of included patients according to their clinical data, death, and mean MGOS score. NA: not applicable; GCS: Glasgow Coma Scale; WFNS: World Federation of Neurosurgeons; CT: computed tomography; MGOS: Modified Glasgow Outcome Scale

Variable	No.	(%)
Comorbidity		
Yes	28	58.3
No	18	37.5
NA	2	4.2
If yes, what disease:		
Hypertension	18	37.4
Diabetes mellitus	8	16.7
Cancer	2	4.2
Cardiovascular disease	7	14.6
Blood disorders	4	8.3
Thyroid disorders	1	2.1
Others	4	8.3
Initial GCS score	12.62 ± 3.56	
WFNS score at admission	2.19 ± 1.54	
Presence of aneurysm		
NA	6	12.5
No	21	43.8
Yes	21	43.8
Location of aneurysm		
NA	2	4.2
Anterior communicating artery	12	25
Bilateral inferior frontal (predominantly left side), Left temporal lobe, and left Occipital lobe	1	2.1
Internal carotid artery	2	4.2
Right middle cerebral artery	3	6.3
Right posterior communicating artery	1	2.1
Surgical or interventional procedures		
NA	2	4.2
Brain CT scan and angiogram, percutaneous tracheostomy	1	2.1
Brain tumor resection, post cranial fossa decompression, brain biopsy (Burr hole)	1	2.1
Cerebral angiogram	3	6.3
Cerebral angiography with coiling	11	22.9
Clipping of cerebral aneurysm	5	10.4
CT scan of the brain	3	6.3
Endoluminal repair of the aneurysm	1	2.1
Insertion of external ventricular drain, and open tracheostomy	1	2.1
Previous ethmoidectomy for invasive fungal sinusitis	1	2.1
Reinforcement of cerebral aneurysm	1	2.1
Subdural hemorrhage evacuation	1	2.1
None	16	33.3
Death		
No	38	79.2
Yes	10	20.8
MGOS	3.58 ± 1.67	

**Figure 1:**
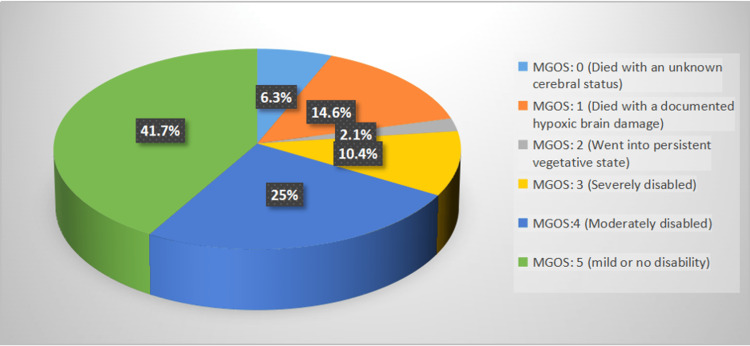
Distribution of the included patients according to MGOS grade. MGOS: Modified Glasgow Outcome Scale

As shown in Figure 1, 14.6%, 2.1%, 10.4%, 25%, and 41.7% of the patients were assigned MGOS Grades 1, 2, 3, 4, and 5 [A1] [A2], respectively. None of the patients were assigned MGOS grade 0. As shown in Table 6, female patients had significantly higher mean MGOS scores compared with male patients (4.16 ± 1.5 vs. 3.23 ± 1.69; p ≤ 0.05). Further, mean MGOS score showed a non-significant relationship with the patients’ nationality, marital status, and smoking history (p > 0.05).

**Table 6:** Relationships between mean MGOS score and patients’ demographic data and smoking history. N.B.: *=Mann–Whitney test **=Kruskal–Wallis test; NA: not applicable; MGOS: Modified Glasgow Outcome Scale; SD=standard deviation The asterisks indicate the non-parametric test used for the statistical analysis.

Variable	MGOS (Mean ± SD)	Test	p-value
Gender			
Female	4.16 ± 1.5	2.3*	0.021
Male	3.23 ± 1.69		
Nationality			
Saudi	3.41 ± 1.83	2**	0.556
Non-Saudi	3.6 ± 1.6		
NA	3.21 ± 1.7		
Marital status			
NA	4.27 ± 1	2**	0.129
Married	3.23 ± 1.7		
Single	3.72 ± 2		
Smoking history			
NA	4.33 ± 1	2**	0.256
No	3.53 ± 1.74		
Yes	2.85 ± 1.86		

As shown in Table 7, patients who died had significantly lower mean MGOS scores (p ≤ 0.05). Moreover, mean MGOS score showed a non-significant relationship with the patients’ clinical characteristics (p > 0.05).

**Table 7:** Relationships between mean MGOS score and patients’ clinical data and death status. N.B.: *=Mann–Whitney test **=Kruskal–Wallis test; NA: not applicable; MGOS: Modified Glasgow Outcome Scale; SD=standard deviation The asterisks indicate the non-parametric test used for the statistical analysis.

Variable	MGOS (Mean ± SD)	Test	p-value
Comorbidity			
Yes	3.28 ± 1.8	2**	0.356
No	4 ± 1.45		
NA	4 ± 1.41		
If yes, what disease:			
Hypertension	3.44 ± 1.68	0.74*	0.454
Diabetes mellitus	2.87 ± 2.23	0.33*	0.376
Cancer	4.5 ± 0.7	0.51*	0.574
Cardiovascular disease	3.28 ± 2.28	0.83*	0.841
Blood disorders	4.75 ± 0.5	0.11*	0.145
Thyroid disorders	5 ± 0.001	0.28*	0.417
Other	2.75 ± 2.06	0.36*	0.416
Presence of aneurysm			
NA	3.16 ± 1.83	2**	0.659
No	3.47 ± 1.77		
Yes	3.81 ± 1.56		
Location of aneurysm			
NA	4.5 ± 0.7	5**	0.679
Anterior communicating artery	3.33 ± 1.87		
Bilateral inferior frontal (predominantly left side), left temporal lobe, and left occipital lobe	4 ± 0.001		
Internal carotid artery	5 ± 0.001		
Right middle cerebral artery	4.33 ± 1.15		
Right posterior communicating artery	4 ± 0.001		
Death			
No	4.34 ± 0.81	5.06*	<0.001
Yes	0.7 ± 0.48		

**Figure 2:**
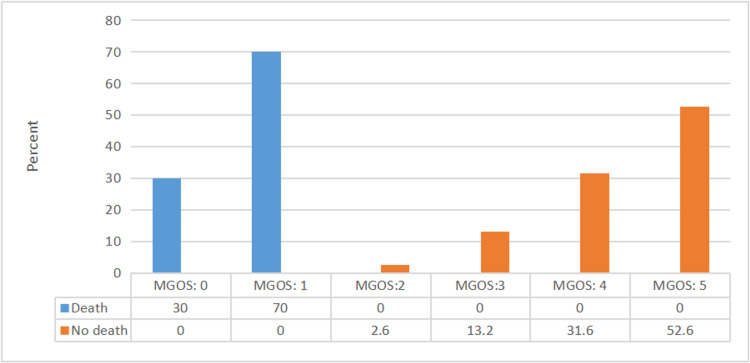
Relationship between MGOS grades and death. N.B.: (χ2=48, p-value ≤ 0.001); MGOS: Modified Glasgow Outcome Scale

**Figure 3:**
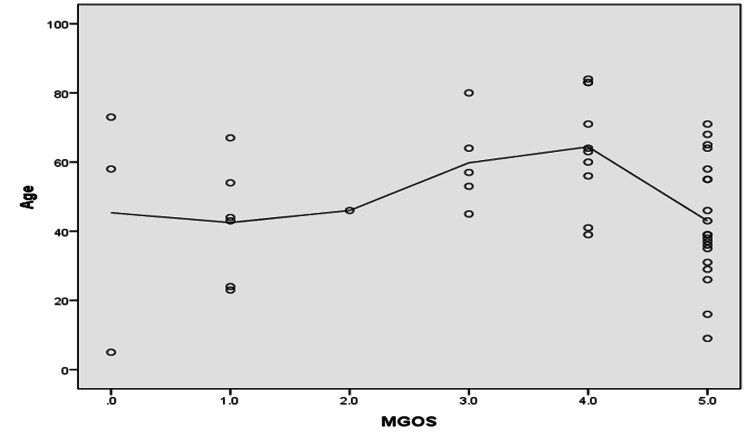
Spearman’s correlation analysis between MGOS score and age. N.B.: r=−0.17, p-value=0.24; MGOS: Modified Glasgow Outcome Scale

**Figure 4:**
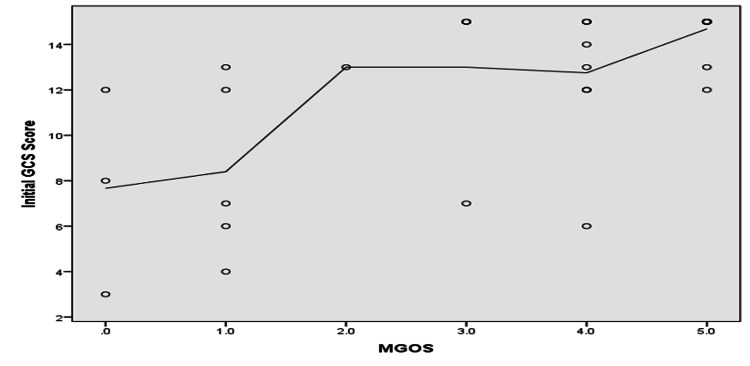
Spearman’s correlation analysis between MGOS score and initial GCS score. N.B.: r=0.68, p-value ≤ 0.001; MGOS: Modified Glasgow Outcome Scale, GCS: Glasgow Coma Scale

**Figure 5:**
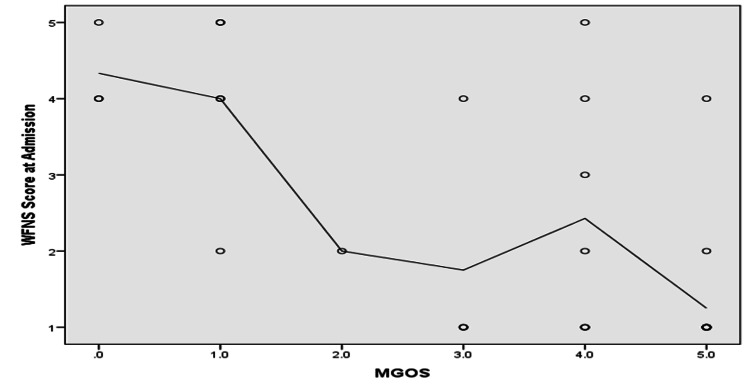
Spearman’s correlation analysis between MGOS score and WFNS score at admission. N.B.: r=−0.67, p-value ≤ 0.001; MGOS: Modified Glasgow Outcome Scale; WFNS: World Federation of Neurosurgeons

As shown in Figure 2, patients with MGOS grades 1 and 5 comprised the highest percentage of dead and living patients, respectively (p ≤ 0.05). As shown in Figures 3-5, MGOS score showed a non-significant negative correlation with age (r=−0.17, p=0.24). In contrast, MGOS score showed significant positive and negative correlations with the baseline GCS and WFNS scores at admission, respectively (r=0.68 and r=−0.67, respectively, both p<0.001).

## Discussion

Our findings demonstrated that comorbidities significantly affected the QoL of patients with SAH. Female gender showed a negative effect on the outcomes of SAH patients, which agrees with the findings of both Meyer et al. [14] and Katati et al. [13], and is consistent with a previous hypothesis that compared with men, women have an increased risk of SAH, with a higher occurrence of multiplicity (i.e., strokes in multiple brain regions) [18]. MGOS scores were not correlated with marital status or nationality. Additionally, MGOS scores were not correlated with smoking history. However, several studies have reported an unexpected protective effect of smoking on SAH outcomes - even though cigarette smoking is considered among the most significant risk factors for cerebral aneurysm [19]. Slettebø et al. found that smokers had a lower 30-day mortality than that of nonsmokers, and the functional outcome of the former was not inferior to that of the latter [20].

Notably, we observed a correlation between the initial GCS score at admission and patient outcomes. MGOS score was negatively correlated with mortality rate, which is consistent with previous reports. A low GCS score at admission was the most powerful predictor of poor outcomes in SAH patients, which is consistent with previous reports that poor neurological status on admission is generally associated with poor outcomes after SAH [21]. This predictive power was also corroborated by Drake et al. [22], who found that the GCS score at the admission of SAH patients is a more dependable aneurysmal SAH grading system.

WFNS score on admission was negatively correlated with MGOS score, where the higher the WFNS score, the poorer the patient’s condition, and therefore, the worse the outcome. In a previous study on the timing of grading aneurysmal SAH patients, Giraldo et al. reported that the weakest predictor of poor outcome was WFNS score determined at admission [23]. This difference can be attributed to the study form, which focused only on aSAH, and they were able to apply standardized intervention for the patients (e.g., microsurgical clipping, endovascular occlusion, and medical treatment), as compared to our interventions shown in Table 2. The patients were also closely followed up for six months without having any intervening or disruptive factors.

The current study did have some potential limitations. First, the sample size was small, which may have created a selection bias. Second, we were not able to collect data on several other variables due to the retrospective nature of the study.

## Conclusions

Considerable progress has been made in relation to QoL after SAH, and our findings certainly add to this growing body of literature. As few studies of this type have been conducted in the western province of Saudi Arabia, this study creates opportunities to further explore and understand QoL. Moreover, despite considering the WFNS and MGOS unequivocal grading systems, correlating them with various patient factors provided a more sensible and realistic description of the QoL.
